# Rotavirus Induces Epithelial–Mesenchymal Transition Markers by Transcriptional Suppression of miRNA-29b

**DOI:** 10.3389/fmicb.2021.631183

**Published:** 2021-02-18

**Authors:** Urbi Mukhopadhyay, Anwesha Banerjee, Mamta Chawla-Sarkar, Anupam Mukherjee

**Affiliations:** ^1^Division of Molecular Virology, ICMR-National Institute of Cholera and Enteric Diseases, Kolkata, India; ^2^Division of Virology, ICMR-National AIDS Research Institute, Pune, India

**Keywords:** microRNA, trans-suppression, rotavirus, EMT, miR-29b

## Abstract

Acute gastroenteritis (AGE) is a serious global health problem and has been known to cause millions of infant deaths every year. Rotavirus (RV), a member of the *Reoviridae* family, still majorly accounts for the AGE in children below 5 years of age in India and worldwide. The involvement of miRNAs in the pathogenesis of RV has been suggested to be of the proviral as well as the anti-viral nature. miRNAs that promote the RV pathogenesis are capable of targeting the cellular components to evade the host anti-viral strategies. On the other hand, miRNAs with anti-rotaviral properties are themselves incapacitated during the progression of the infection. The exploitation of the epithelial–mesenchymal transition (EMT) as a pro-rotaviral strategy has already been identified. Thus, miRNAs that proficiently target the intermediates of the EMT pathway may serve as anti-viral counterparts in the RV–host interactions. The role of microRNA-29b (miR-29b) in the majority of human cancers has been well demonstrated, but its significance in viral infections is yet to be elaborated. In this study, we have assessed the role of miR-29b in RV-induced EMT and RV replication. Our study on miR-29b provides evidence for the recruitment of RV non-structural protein NSP1 to control the trans-repression of miR-29b in a p53-dependent manner. The trans-repression of miR-29b modulates the EMT pathway by targeting tripartite motif-containing protein 44 (TRIM44) and cyclin E1 (CCNE1). SLUG and SNAIL transcription repressors (downstream of TRIM44 and CCNE1) regulate the expression of E-cadherin, an important marker of the EMT. Also, it is established that ectopic expression of miR-29b not only constrains the EMT pathway but also restricts RV replication. Therefore, miR-29b repression is a crucial event in the RV pathogenesis. Ectopic expression of miR-29b displays potential anti-viral properties against RV propagation.

## Highlights

– The study focuses on the significance of microRNA-29b (miR-29b) in rotavirus (RV) replication and spread.
– RV recruits viral protein NSP1 to direct the trans-repression of miR-29b.
– miR-29b trans-suppression is p53-mediated.
– Tripartite motif-containing protein 44 (TRIM44) and cyclin E1 (CCNE1), the targets of miR-29b, are intermediates in the epithelial–mesenchymal transition (EMT) pathway.
– Ectopic miR-29b expression restricts the EMT pathway along with RV replication.

## Introduction

Rotavirus (RV), a major cause of morbidity and mortality in children due to RV-induced acute gastroenteritis (AGE), was associated with more than 200,000 deaths among children worldwide, and has been still accounted for 40% of the total diarrheal mortality in Southeast Asia (Tate et al., 2016; Lestari et al., 2020). As a member of the *Reoviridae* family, RVs are non-enveloped, RNA viruses. They are 70 nm in diameter and their genome consists of segmented, double-stranded RNA. The RNA segments encode for either the RV-structural proteins (VP1–VP4, VP6, and VP7) or non-structural proteins (NSP1–NSP5) (Estes and Kapikian, 2007). The NSP1 protein of RV comprises a recognized N terminal RING domain. The RING domain is involved in the specific interaction with the 5’ termini of the RV mRNAs to prevent the activation of the RNA sensors in the cell (Hua et al., 1994). The C terminus of NSP1 variably interacts with some of the host proteins to direct their degradation. This degradation takes place in a proteasome-dependent manner where NSP1 acts as an E3 ubiquitin ligase (Graff et al., 2007). NSP1 is capable of degrading p53 which is a transcription factor (Bhowmick et al., 2013). Being an essential regulator of cellular processes, p53 plays a pivotal role in controlling the genes involved in cell cycle progression, epithelial–mesenchymal plasticity, apoptosis, and cellular metastasis (Ren et al., 2013).

miRNAs are non-coding RNAs that have an average length of 22 nucleotides and are regulators of mRNA transcripts. The primary miRNAs (pri-miRNAs) are generally transcribed from DNA sequences and acted upon by a complex of proteins into the precursor miRNAs (pre-miRNAs) and the mature miRNAs (Ha and Kim, 2014). The mature miRNAs regulate the gene expression by interacting with the 3′UTR or 5′UTR, or in some cases, the coding or the promoter regions of the mRNA targets (Broughton et al., 2016). These regions, also known as the miRNA regulatory elements (MREs), are not completely complementary to the miRNA. The miRNA–MRE interaction occurs via the 5′ seed region (nucleotides 2–8) of the miRNA (Ellwanger et al., 2011; Xu et al., 2014). As with protein-coding genes, the miRNA expressions are controlled by the promoter-transcription factor interactions. p53, a stress-induced transcription factor, can increase the expressions of miR-34 and miR-107 to facilitate the cell cycle arrest and induce apoptosis (He et al., 2007). Also, p53 directly binds to the miRNA promoter and transcriptionally activates the miRNA to regulate the EMT pathway (Chang et al., 2011). p53 has been found to be hijacked in many disease conditions, such as the one found in this study, to manipulate miRNA-mediated regulation of proviral proteins.

The epithelial–mesenchymal transition (EMT) is a dynamic event and is involved in wound healing and developmental processes (Lambert et al., 2017). It results in the conversion of the epithelial cells to mesenchymal cells and encourages cell migration and invasion (Moustakas and de Herreros, 2017). When the EMT is triggered, the junctions connecting one cell to the other are de-structured or destroyed (Huang et al., 2012). The destabilization is accompanied by cleavage of the epithelial (E)-cadherin at the cell membrane and eventual degradation (Yilmaz and Christofori, 2009). The progression of EMT represses the expression of the junction proteins stabilizing the loss of the epithelial junctions. Bacterial and viral infections induce the EMT pathway in the epithelial cells. Like the RVs, hepatitis C virus (HCV) is an RNA virus. HCV is capable of inducing the EMT pathway via the non-structural HCV protein (NS)-5A-mediated enhanced phosphorylation of Akt (protein kinase B), followed by activation of β-catenin. Hepatocellular EMT is also instigated by the HCV NS4A protein (Akkari et al., 2012; Hu et al., 2017). Another RNA virus, the SARS-CoV-2 virus, is proficient in triggering the EMT pathway, which causes the loss of expression of the ACE2 receptor (Stewart et al., 2020). As intestinal epithelial cells are the major targets of RV, induction of EMT is clearly observed in RV infection. The EMT pathway could, however, be blocked by hsa-miR-142-5p, an epithelial cell miRNA (Chanda et al., 2016). Also, in HCV infection of hepatocytes, depletion of miR-122 has shown to promote EMT-like changes through the activation of β-catenin (Liang et al., 2016). Thus, the role of miRNAs in the alterations of the EMT pathway has been known in some viral infections.

Here, we have identified microRNA-29b (miR-29b) as a crucial suppressor of the EMT pathway induced in the RV infection. We further demonstrated that the expression of miR-29b was significantly reduced through trans-suppression of p53 by RV-NSP1. The mechanisms adopted by RV to repress this miRNA are directly correlated with the efficient viral infection. The direct role of miR-29b in decreasing RV replication has also been investigated. Therefore, these data suggest that the ectopic supplement of miR-29b may have therapeutic potential in RV infection.

## Materials and Methods

### Cells and Viruses

Human epithelial colorectal adenocarcinoma cells (Caco-2), African Green monkey fetal kidney epithelial cells (MA104), and human embryonic kidney cell (293T) were cultured either in complete growth medium (EMEM) or minimum essential medium (MEM) supplemented with 10–20% heat-inactivated fetal bovine serum, 0.1 mM of non-essential amino acids, 2 mM sodium pyruvate, and 1% antibiotic–antimycotic, and maintained at 37°C humidified incubator with 5% CO_2_. The cell culture adapted simian RV strain SA11 (H96), human RV strains Wa, KU, and bovine RV strains A5-13, and NSP1 mutant A5-16 were used in this study. The mutant A5-16 RV has a 500-nucleotide deletion in the DNA sequence encoding the RING domain of NSP1, which is followed by a non-sense codon rendering the A5-16 NSP1 non-functional (Taniguchi et al., 1996). The viral strains were propagated in the MA104 cells. Extracted viral preparations were titrated and calculated by plaque assay as described earlier (Estes and Kapikian, 2007; Bagchi et al., 2010). The viruses were activated with acetylated trypsin (10 g/ml) at 37°C before infection, and the cells were incubated with 3 moi (multiplicity of infection) of the preactivated virus for 45 min at 37°C. To develop UV-inactivated RV, the virus was pretreated with 40 μg/ml psoralen AMT for 15 min followed by irradiation by long-wave UV-light (365 nm) for 2 h under ice-cold condition (Mukhopadhyay et al., 2019). All the cell culture, virus cultivation, and infection protocols for the experiments were carried out under Biosafety Level 2 conditions.

### Cell Viability Assay

To check the cytotoxicity of chemicals, MA104 or Caco-2 cells were transfected with scrambled miRNA or mimic or inhibitor of miR-29b or inhibitor of p53-mediated apoptosis, Pifithrin-α (PFT-α), and proteasome inhibitor MG132 in 96-well plates. By post-transfection or treatment, the cells were treated with the reagent solution in a serum-free medium within a humidified 5% CO_2_ incubator maintaining 37°C atmosphere, following the manufacturer’s instruction (Promega: G3581). The viable cells were measured spectrophotometrically at 490 nm using a plate reader (Thermo Fisher Scientific). The percent viability was calculated considering 100% viability for mock control at similar endpoints.

### RNA Quantitation and Reverse Transcription-Quantitative PCR

Total cellular RNA was isolated using TRIzol reagent (Invitrogen: 15596018) according to the manufacturer’s instructions. cDNA was prepared by using miR-29b or U6-specific primers with a TaqMan microRNA reverse transcription kit (Applied Biosystem: 4366597). Superscript III reverse transcriptase (Invitrogen: 18080-051) and random hexamer was used for tripartite motif-containing protein 44 (TRIM44), cyclin E1 (CCNE1), RV-NSP4, RV-VP6, and GAPDH. Real-time PCR reactions were performed using TaqMan universal PCR master mix and 6-carboxyfluorescein (FAM)-MGB probes (Applied Biosystem) for quantification of miR-29b (Assay ID: 002165). U6 (Assay ID: 001973) was used as an endogenous control for microRNA expression. For gene expression studies, SYBR^®^ Green master mix (Applied Biosystems, United States) with specific primers for TRIM44, CCNE1, RV-NSP4, and RV-VP6 was used for quantitation (Supplementary Data 1). GAPDH was used as an endogenous control for gene expression. The relative expression levels were normalized to the endogenous control by using the 2^–ΔΔCT^ formula (ΔΔC_T_ = ΔC_T_ of the sample −ΔC_T_ of the untreated control).

### Plasmid Construction

The full-length (P0) or different deletion mutants (P1–P3) of miR-29b promoter fragments were amplified from 293T genomic DNA by using specific primers (Supplementary Data 1). The fragments were digested with *Mlu*I and *Hin*dIII and cloned into the pGL3-Basic luciferase vector (Promega). The 3′UTR luciferase reporter constructs of TRIM44 and CCNE1 were prepared by cloning the PCR-amplified human TRIM44 and CCNE1 mRNA 3′UTRs into the *Mlu*I/*Hin*dIII site of the pMIR-REPORT miRNA expression luciferase reporter plasmid (Ambion: AM5795). Mutant 3′UTRs of TRIM44 and CCNE1 were used as a control in parallel; ∼120 bp of an upstream and downstream flanking pre-miR-29b sequence was obtained from the UCSC genome browser, and amplified and ligated into *Xho*I/*Kpn*I site of the pmR-ZsGreen1 mammalian expression vector (Takara Bio, United States) designed to constitutively express the microRNA of interest. The p53 vector set containing pCMV-p53 was purchased commercially (Clontech, United States). The non-structural proteins of RV (NSP1-5) and the truncated mutants of RV-NSP1 were cloned in pcDNA6B, and the different domains of p53 were cloned in the pFLAG-CMV vector as described earlier (Bhowmick et al., 2013).

### Transfection of Cells

MA104 and 293T cells were cotransfected with the plasmid containing miR-29b promoter (200 ng) and equal amounts of plasmid DNA containing RV-NSP1, p53, or the truncated mutants of RV-NSP1 or p53 by using Lipofectamine 3000 Reagent (Thermo Fisher Scientific: L3000001). Mimics and inhibitors of hsa-miR-29b were transfected (final concentration 10–40 nM) in 293T and Caco-2 cells with Lipofectamine^®^ RNAiMAX Reagent (Thermo Fisher Scientific: 13778100) and TransfeX^TM^ Transfection Reagent (ATCC^®^ ACS-4005^TM^), according to manufacturer’s recommendations.

### Luciferase Reporter Assays

The pGL3-Basic luciferase vector containing miR-29b promoter fragments (P0–P3) and the pMIR-REPORT miRNA expression luciferase reporter plasmid containing 3′UTRs of TRIM44 or CCNE1 were transfected into cells, to determine the miR-29b promoter activity in the presence of RV-NSP1 and/or p53 constructs and for target validation in the presence of scrambled-miR or different doses of mimic miR-29b, respectively. The firefly luciferase activities were recorded by Dual-Luciferase^®^ Reporter Assay System (Promega: E1960) after normalization to the expression of control Renilla luciferase.

### Immunoblot Analysis

Cells were washed with phosphate-buffered saline and lysed in RIPA buffer, and the concentration of proteins was quantitated by Pierce^TM^ BCA Protein Assay Kit (ThermoFisher Scientific: 23225). Whole-cell lysates were subsequently subjected to polyacrylamide gel electrophoresis and transferred onto polyvinylidene difluoride (PVDF) membranes. The membranes were blocked with 5% non-fat dried milk and incubated with specific antibodies. The membranes were probed with antibodies to TRIM44 (#ab236422), CCNE1 (#ab238081) (AbCam), E-Cadherin (#3195), N-Cadherin (#4061), Snail (#3879), Slug (#9585) (Cell Signaling), RV-VP6 (HyTest: 3C10), and RV-NSP1 or RV-NSP3 (kind gift from Prof. Koki Taniguchi). Primary antibodies were detected with horseradish peroxidase (HRP)-conjugated secondary antibodies (Pierce, United States) and enhanced chemiluminescence (ECL) substrate (Millipore, United States) within ChemiDoc Imaging System (Bio-Rad) or onto BioMax Film (Kodak). All membranes were reprobed with GAPDH (Santa Cruz: sc-47724HRP) as an internal loading control. Band intensities were measured using ImageJ software v1.53a, normalized to loading control, and represented as relative fold changes. The immunoblots used in this manuscript were checked for their compliance with the digital image and integrity policies.

### Immunofluorescence

MA104 cells, grown on glass coverslips (18 mm square, no. 1; Blue Star) and transfected and/or infected with pmR-ZsGreen1-pre-miR-29b and RV, were fixed with paraformaldehyde (4% w/v in PBS) and further processed as described earlier (Mukhopadhyay et al., 2019). Cells were incubated with RV-NSP5 (1:200; Rabbit monoclonal; a kind gift from Prof. Koki Taniguchi) diluted in blocking solution at 4°C. After overnight incubation, the cells were washed and further treated with Rhodamine-conjugated goat-anti-mouse (ThermoFisher Scientific: 31660) secondary antibodies for 2 h in the dark in a humidified 37°C incubator. Nuclei were visualized after incubation with Vectashield containing 4′,6′-diamidino-2-phenylindole (DAPI) and mounted on microscope slides. Mounted slides were examined under a Zeiss Axioplan confocal microscope (63x oil immersion). The images were captured and processed using ZEN Blue software v3.1 (Carl Zeiss Microscopy GmbH, Jena, Germany) and saved as 24-bit tagged JPG images in RGB-format. For comparison between different samples, images were collected during a single session at identical excitation and detection settings.

### Ago2-RNA Co-immunoprecipitation

The RV RNA and miR-29b complex were precipitated as described previously (Mukhopadhyay et al., 2019). Briefly, miR-29b-transfected Caco-2 cells were lysed with cell lysis buffer, clarified, and incubated with anti-Ago2 mAb (Cell Signaling: 2897) or isotype control IgG2a at 4°C for overnight. The antibody-coupled lysates were then mixed with Protein G Sepharose beads (GE Healthcare: 17061802) for 4 h. Ago2 co-immunoprecipitated RNA was isolated using the RNeasy Mini Kit (Qiagen: 74104) after washing the beads four times. TRIM44 and CCNE1 expressions were quantified by qRT-PCR using SYBR^®^Green chemistry and miR-29b expression by TaqMan probe chemistry as described earlier.

### Statistical Analysis

The results are presented as means from at least two or three independent experiments ± standard deviations or standard error of the mean. The *p*-value ≤ 0.05 is considered to be statistically significant for all experiments and presented as asterisks (^∗^*p* ≤ 0.05; ^∗∗^*p* ≤ 0.01; ^∗∗∗^*p* ≤ 0.001). Statistical significance of the results has been analyzed using Student’s *t*-test. The figures having more than two experimental groups were analyzed by pairwise ANOVA using GraphPad Prism 8.4.3.

## Results

### RV Infection Leads to Suppression of miR-29b Expression

**FIGURE 1:**
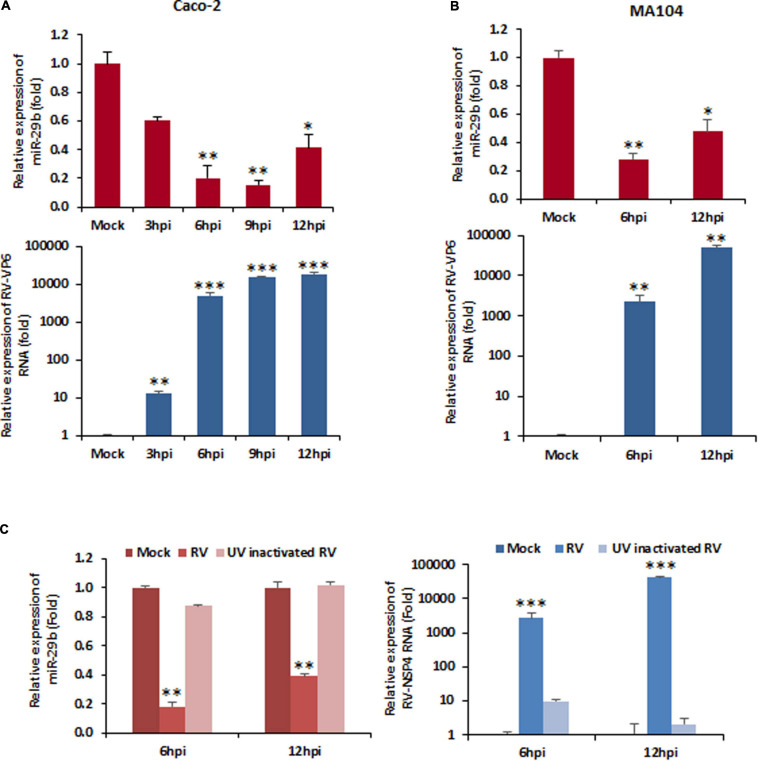
Rotavirus (RV) infection downregulates miR-29b expression. Cells were mock treated or infected with RV-SA11 for indicated time points. Total RNA from mock- or virus-infected cells was extracted. **(A)** Expression of miR-29b was analyzed in RV-infected Caco2 cells by qRT-PCR and normalized to the expression of U6 snRNA (upper panel). RV-VP6 RNA level was measured in the same cell lysates and plotted as relative RNA level in comparison to mock-infected cells after normalization to the expression of GAPDH (lower panel). **(B)** miR-29b expression was analyzed in RV-infected MA104 cells (upper panel), and level of viral RNA was measured by RV-VP6 expression in the same cell lysates. **(C)** Relative expression of miR-29b was analyzed by qRT-PCR in UV-inactivated RV-SA11-infected MA104 cells (left panel). RV-NSP4 RNA level was measured by qRT-PCR in UV-inactivated RV-SA11-infected cells (right panel). Results are presented as the means and standard deviations from at least four independent experiments. ^∗^*p* ≤ 0.05; ^∗∗^*p* ≤ 0.01; ^∗∗∗^*p* ≤ 0.001.

miRNAs have cell-specific functions, pertaining to the fact that they may have more than one target and maybe the target in more than one pathological condition. Since RV infection alters the function of mature enterocytes lining the small intestine and miR-29b is known for mucosal atrophy in the same area targeting several mRNAs encoding proteins, the modulation of miR-29b in RV-infected epithelial cells was examined. Mock-infected or RV-SA11-infected Caco2 cells were monitored for changes in expression of miR-29b at different time intervals of infection (Figure 1A). The expressions of RV-VP6 (intermediate capsid protein of RV) were checked in the same cell lysates as evidence of efficient RV infection at these time points. The expression of miR-29b was significantly downregulated by approximately fivefold at 6 h post-infection (hpi) when RV-VP6 expression was high. The same result was obtained with RV-SA11 infection of MA104 cells (Figure 1B). The expression of the miR-29b was normalized with the expression of U6 snRNA, while that of RV-VP6 was normalized with GAPDH and plotted as a relative fold change in RNA expression compared to the mock-infected samples. To confirm that active, replicating RV is responsible for the downregulation of miR-29b in target epithelial cells, MA104 cells were infected with UV-inactivated RV-SA11 and analyzed for the relative expression of miR-29b. RV-NSP4 expression was checked in these samples to mark efficient RV infection (Figure 1C). miR-29b suppression was not observed in the UV-inactivated RV-SA11 samples compared to active RV infection where RV-NSP4 expression was high. Altogether, these results show that RV replication suppresses miR-29b expression in epithelial cells.

### Rotavirus Employs Non-structural Protein 1 to Downregulate miR-29b Expression

**FIGURE 2:**
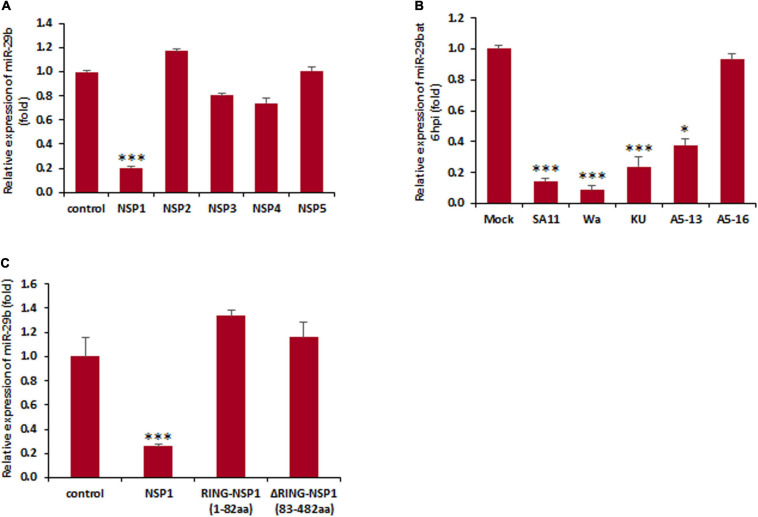
Rotavirus-induced miR-29b dysregulation is NSP1 dependent. **(A)** MA104 cells were transfected with the plasmid DNA encoding RV non-structural proteins. Relative expression of miR-29b was measured after 48 h of post transfection. **(B)** Caco2 cells were infected with different RV strains SA11 or Wa or KU or A5-13 or A5-16 or kept mock infected. Expression of miR-29b was analyzed at early stage of infection at 6 hpi. **(C)** MA104 cells were either mock transfected or transfected with pcDNSP1, pcDRING-NSP1, or pcDΔRING-NSP1, and the expression of miR-29b was examined. All results are presented as the means and standard deviations from three independent experiments. ^∗^*p* ≤ 0.05; ^∗∗∗^*p* ≤ 0.001.

The knowledge that replicating RV and not UV-inactivated RV is responsible for the dysregulation of miR-29b suggested the involvement of an actively expressed viral protein in the modulation of miR-29b expression. In order to recognize the viral protein actively responsible for this downregulation, MA104 cells were transfected with NSP1-5 individually and analyzed for the relative change in miR-29b expression at 48 h post-transfection by qPCR (Figure 2A). The expression level of miR-29b in NSP1-transfected cells was suppressed by ∼five folds, as was observed in our earlier experiments with the replication-competent virus. The transfection efficiency of NSP1-5 proteins has been confirmed by immunoblot analysis (Supplementary Data 2). This dysregulation of miR-29b is not restricted to the simian RV SA11 strain of RV and could be observed while infection with other human and bovine RV strains (Wa, KU, and A5-13) at 6 hpi (Figure 2B). Our claim of NSP1-dependent miR-29b suppression was found to be conclusive when the expression of this miRNA was unaltered in NSP1-mutated A5-16 RV, the NSP1 mutant variant of bovine RV strain. The same cell lysates were checked for RV-VP6 expression as evidence of efficient RV infection (Supplementary Data 3).

We further attempted to identify the specific domain of NSP1 responsible for the suppression of miR-29b. To establish this, the cells were transfected with or without the RING domain cloned into the pcDNA. MA104 cells were mock transfected or pcDNSP1 (wtNSP1), pcDRING-NSP1 (1–82 amino acids), or pcDΔRING-NSP1 (83–482 amino acids) transfected and assessed for the relative change in expression levels of miR-29b (Figure 2C). It was interesting to note that though the suppression was observed in the wild-type NSP1-transfected samples, it was not depicted in the others. The transfection efficiency of wtNSP1 or its truncated domains have been confirmed by immunoblot analysis (Supplementary Data 4). Therefore, our results suggest that granting RV-employed NSP1 may be necessary for the suppression of miR-29b expression, not a single domain, but the entire protein may be crucial for this function.

### miR-29b Suppression Is Transcriptionally Regulated by Rotavirus

**FIGURE 3:**
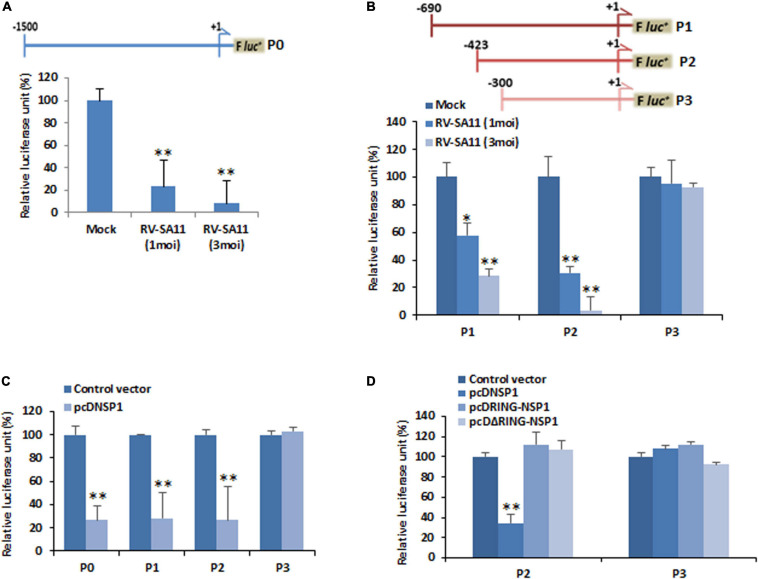
RV transcriptionally downregulates miR-29b. **(A)** Schematic diagram of the miR-29b promoter region (nucleotides –1,500 to +1; P0) cloned into the pGL3-Basic luciferase reporter plasmid (upper panel). MA104 cells were transfected with the miR-29b–luciferase reporter construct for 48 h followed by infection with RV-SA11. Relative luciferase activity was measured after 6 h of post infection (lower panel). **(B)** Deletion mutants of the miR-29b promoter construct (nucleotides −690 to +1; P1, −423 to +1; P2, and −300 to +1; P3: upper panel) were transfected into MA104 cells followed by infection with RV-SA11. The effect of RV on these mutants was examined by luciferase reporter assay (lower panel). **(C)** 293T cells were cotransfected with the miR-29b promoter constructs (P0, P1, P2, or P3) and RV-NSP1. The effect of RV-NSP1 on these mutants was examined by luciferase reporter assay. **(D)** Cells were cotransfected with either P2 or P3 miR-29b promoter constructs along with pcDNSP1, pcDRING-NSP1, or pcDΔRING-NSP1, and the relative luciferase units were measured. Data are presented as the means and standard deviations from at least three independent experiments. ^∗^*p* ≤ 0.05; ^∗∗^*p* ≤ 0.01.

Since the expression of miR-29b was found to be suppressed, we examined the contribution of its promoter activity in its suppression. The miR-29b promoter sequence was cloned into pGL3 luciferase reporter plasmid DNA. P0 (nucleotides −1,500 to +1 of miR-29b)-transfected and RV-SA11-infected MA104 cell samples showed a decrease in miR-29b promoter activity, as evidenced by luciferase reporter assay, with increasing moi of RV infection (Figure 3A). This led us to believe that miR-29b was being regulated within this P0 region. A series of deletion mutants of the miR-29b promoter were constructed and transfected in MA104 cells. With an increase in RV infection, where lowered luciferase activity was observed in P1 (nucleotides −690 to +1) and P2 (nucleotides −423 to +1)-transfected samples, the luciferase activity remained unaltered in P3 (nucleotides −300 to +1)-transfected samples post-infection (Figure 3B). These observations indicated that the regulatory region of mature miR-29b was located within 300–423 nucleotides upstream of the pri-miR-29b promoter. Results from co-transfection studies of the mutant promoter constructs with pcDNSP1 complied with our previous results, thus explaining the approximately fivefold decrease in the promoter activity of miR-29b by RV-encoded NSP1 (Figure 3C). As P1 and P2 both had the regulatory region intact, it became clear that promoter activity was situated in the P1 and the P2 construct. Since P2 contained a sub-sequence of P1, further experiments involved the comparison between the P2 and P3 promoter constructs. Co-transfection of either P2 or P3 with pcDNSP1, pcDRING-NSP1, or pcDΔRING-NSP1 revealed that miR-29b promoter activity in P2 construct was lowered only when co-transfected with pcDNSP1 where the entire NSP1 protein is intact (Figure 3D). These results support our earlier observations and add that the two domains of NSP1 work hand-in-hand to manipulate the promoter activity of miR-29b to cause its suppression.

**FIGURE 4:**
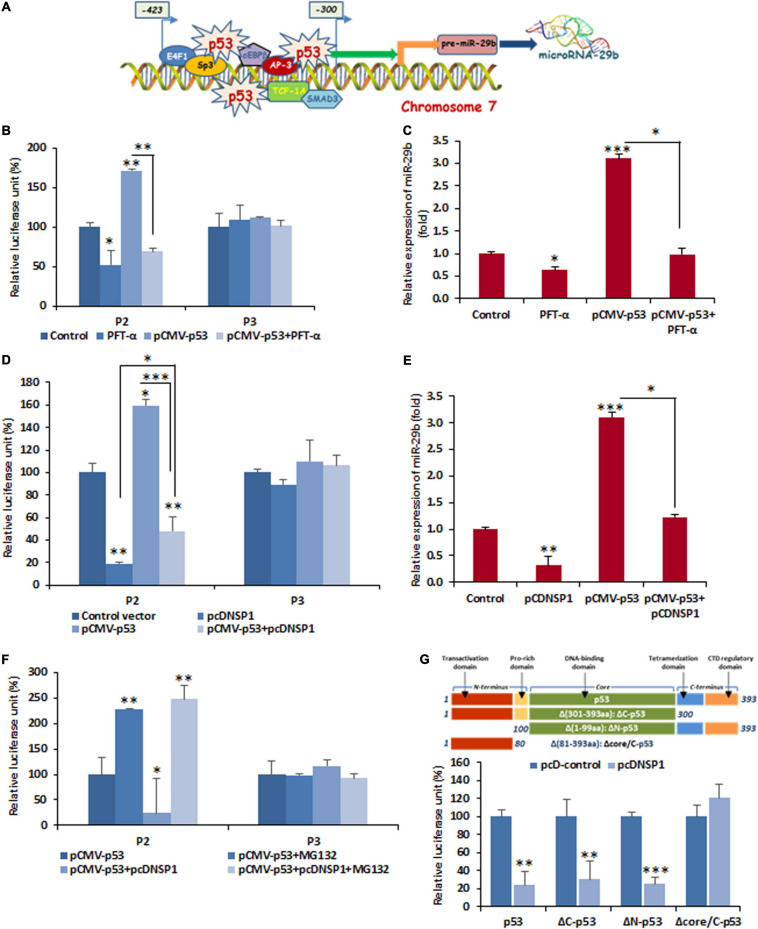
Functional analysis of cis-regulatory elements involved in the suppression of miR-29b by RV NSP1. **(A)** A schematic diagram of predicted transcription factor-binding sites in miR-29b promoter. Transcription factors bind to specific transcription factor-binding sites to enable gene expression, therefore biogenesis of miR-29b. **(B)** Inhibition of p53 is sufficient for miR-29b downregulation. 293T cells were cotransfected with miR-29b promoter construct (P2 or P3), and pCMV-p53 in the presence or absence of Pifithrin-α (PFT-α; 10 μM), followed by luciferase reporter assay. **(C)** Cells were either mock transfected or transfected with pCMV-p53 in the presence or absence of PFT-α, and the expression of miR-29b was examined. **(D)** Cells were cotransfected with P2 or P3 miR-29b promoter construct along with pcDNSP1 and/or pCMV-p53. The effect of NSP1 and/or p53 overexpression on these mutant promoter constructs was examined by luciferase reporter assay. **(E)** Cells were either mock transfected or transfected with pcDNSP1 and/or pCMV-p53, and the expression of miR-29b was examined. **(F)** P2 or P3 promoter constructs were cotransfected with either pcDNSP1 or pCMV-p53 or both in the presence or absence of 10 μM MG132. The activity of miR-29b promoter constructs was examined by luciferase reporter assay. **(G)** Schematic representation of p53 constructs used in this study (upper panel). 293T cells were cotransfected with miR-29b promoter P0 and the different constructs of p53, pCMV-p53, pCMV-ΔC-p53, pCMV-ΔN-p53, and pCMV-Δcore/C-p53 along with pcDNSP1 or empty vector. Relative luciferase activity of miR-29b promoter construct was examined. Results shown here are representative of at least three independent experiments. ^∗^*p* ≤ 0.05; ^∗∗^*p* ≤ 0.01; ^∗∗∗^*p* ≤ 0.001.

Furthermore, this altered promoter activity of miR-29b is an indicator of the transcription factor/s, modulated by RV-encoded NSP1, that has target sites at the cis-regulatory elements located within the P2 and P3 region. *In silico* prediction of transcription factors and their binding sites showed that p53 had three regulatory sites within the promoter region (300–423 nucleotides upstream of the pri-miRNA transcription initiation site) of miR-29b (Figure 4A and Supplementary Data 5). Also, it is already known that RV-NSP1 is capable of exploiting p53 for its pathogenesis (Bhowmick et al., 2013). Co-transfection of P2 and pCMV-p53 in the presence or absence of PFT-α, a p53 inhibitor, revealed that miR-29b expression could be efficiently suppressed by p53 inhibition (Figure 4B). This effect was obviously not observed when the P3 construct was used instead of the P2 construct, since P3 does not contain the cis-regulatory elements required for the binding of p53. Cells transfected with pCMV-p53 had considerably higher miR-29b expression than those exposed to the p53 inhibitor (Figure 4C). We hypothesized that similar results could be observed when the cells would be challenged with NSP1, as RV-NSP1 is a biological inhibitor of p53. Transfection of pcDNSP1, like PFT-α, inhibited the promoter activity in P2 co-transfected cells in the presence or absence of pCMV-p53 (Figure 4D). Similarly, the expression of miR-29b was suppressed in the pcDNSP1-transfected cells with or without pCMV-p53 than mock-transfected or pCMV-p53 alone (Figure 4E). Thus, the regulation of miR-29b expression is p53 mediated. We wondered whether cells treated with MG132, a known proteasome inhibitor, could maintain the steady expression levels of p53 and prevent its degradation even in the presence of NSP1, to recover the expression of miR-29b. When P2 or P3 promoter constructs were co-transfected with pCMV-p53 with pcDNSP1 in the presence or absence of MG132, miR-29b expression was elevated by almost 10 times in MG132-treated NSP1 challenged cells, than untreated (Figure 4F). Therefore, the role of p53 in miR-29b dysregulation leading to RV pathogenesis is indispensable. To shed more light on this role, we used different constructs of p53 (Bhowmick et al., 2014). Cells that received the core domain could demonstrate the inhibition of miR-29b promoter (P0) activity in the presence of NSP1 (Figure 4G). This inhibition was not displayed in the Δcore/C-p53 and pcDNSP1 co-transfected cells. Consequently, it was inferred that RV-NSP1-directed transcriptional suppression of miR-29b is p53-mediated.

### TRIM44 and Cyclin E1 Are the Targets of miR-29b

**FIGURE 5:**
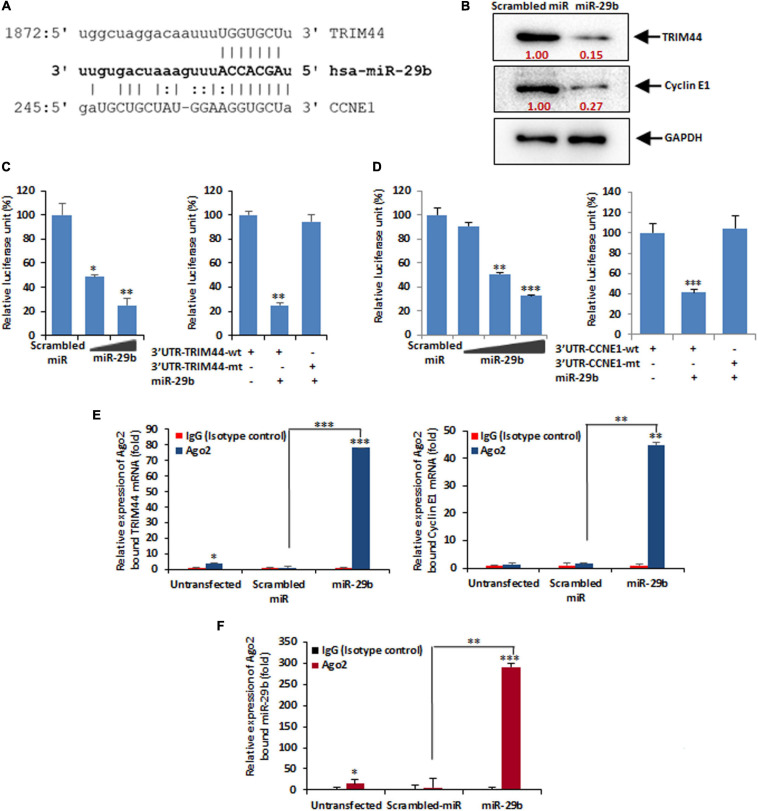
miR-29b targets TRIM44 and cyclin E1. **(A)** *In silico* analysis of the TRIM44 and cyclin E1 or CCNE1 3′UTRs revealed a single putative miR-29b-binding site. The miR-29b target region of TRIM44 (GenBank accession number NM_017583) and CCNE1 (GenBank accession number NM_001238) is indicated. **(B)** Caco2 cells were transfected with mimic miR-29b (40 nM) followed by Western blot analysis using specific antibody against TRIM44 and cyclin E1. Relative fold differences were analyzed after normalization with GAPDH from at least three independent experiments. **(C)** 293T cells were cotransfected with the 3′UTR of TRIM44 cloned into pMIR-REPORT luciferase vector and different doses of the miR-29b mimic (20 or 40 nM). Relative luciferase activity was measured at 48 h post-transfection (left panel). Luciferase activity of the mutant 3′UTR of TRIM44 was not altered in the presence of mimic miR-29b (40 nM) compared to the wild-type 3′UTR (right panel). **(D)** pMIR-REPORT luciferase construct containing the 3′UTR of CCNE1 and different doses of miR-29b mimic (10, 20, or 40 nM) was cotransfected, and relative luciferase activity was measured after 48 h of transfection (left panel). The mutant 3′UTR of CCNE1 was not altered in the presence of mimic miR-29b (40 nM) (right panel). **(E)** Mimic miR-29b-transfected cell lysates were immunoprecipitated with Ago2-specific monoclonal antibody or IgG2a isotype control. RNA was isolated from immunoprecipitates using RNeasy kit. miR-29b bound TRIM44 (left panel) and cyclin E1 (right panel) mRNA expressions were analyzed by qRT-PCR. **(F)** Relative expression of miR-29b from Ago2 immunoprecipitates was analyzed by qRT-PCR. The results are shown as mean and standard deviation from representative of two technical replicates. ^∗^*p* ≤ 0.05; ^∗∗^*p* ≤ 0.01; ^∗∗∗^*p* ≤ 0.001.

Once confirmed that RV-NSP1 is crucial for miR-29b during infection, we aimed at finding the exact function of miR-29b through the identification of its targets. *In silico* studies predicted that TRIM44 and CCNE1 had a common binding site for miR-29b in their 3′UTRs (Figure 5A), thus indicating that TRIM44 and CCNE1 could be the targets of miR-29b, which could be essentially required for RV pathogenesis in the host. To confirm this, the protein levels of TRIM44 and CCNE1 were checked in mimic miR-29b-transfected Caco2 cells (Figure 5B). The expression levels of the two proteins were found to be decreased due to the inhibitory effect arising from miR-29b overexpression as opposed to the scrambled miRNA (miR)-transfected cells. This suggests that TRIM44 and CCNE1 could be the specific targets of miR-29b in epithelial cells. 3′UTR or mutant 3′UTR of TRIM44 and CCNE1 were individually cloned in pMIR-REPORT luciferase vector and co-transfected with different doses of mimic miR-29b. Elevated luciferase signal in mutant 3′UTR-transfected samples clarifies that miR-29b requires the 3′UTR of these targets to suppress their expression (Figures 5C,D), thus settling that miR-29b may be directly interacting with these targets through their 3′UTRs. The direct interactions of miR-29b with its targets were brought to light by the co-immunoprecipitation studies, where mimic miR-29b-transfected, argonaute-2 (Ago-2) immunoprecipitated samples were checked for the presence of TRIM44 and CCNE1 mRNA (Figure 5E). Ago-2 is a component of the RNA-induced gene silencing complex which is required by miR-29b to suppress target expression. Thus, when Ago-2 was immunoprecipitated, TRIM44 and CCNE1 mRNA could be detected with qPCR in the presence of miR-29b. IgG2a Isotype control was included to indicate that the interactions were specific. Also, miR-29b was found associated with Ago-2 along with TRIM44 and CCNE1 (Figure 5F), suggesting that Ago-2, miR-29b, TRIM44, and CCNE1 are in close association with each other. These results together established that miR-29b directly binds to the 3’UTRs of TRIM44 and CCNE1 to suppress their expression, an event that is possibly hijacked by RV to expand its infection in the host.

### Tripartite Motif-Containing Protein 44 and Cyclin E1 Facilitate the Epithelial–Mesenchymal Transition Pathway During Rotavirus Infection

**FIGURE 6:**
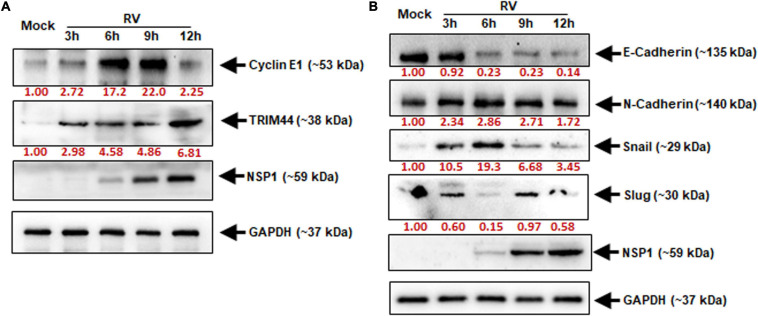
Elevated TRIM44 and cyclin E1 facilitate cellular EMT markers during RV infection. Caco2 cells were infected with RV-SA11 at indicated time points. **(A)** The expressions of cyclin E1 and TRIM44 were examined by immunoblot analysis. GAPDH was used as an internal control for obtaining the expression levels after normalization. NSP1 expression was checked to ensure virus infection. **(B)** Expressions of E-Cadherin, N-Cadherin, Snail, and Slug were checked in the same cell lysates by immunoblotting. Virus infection was confirmed by analyzing NSP1 expression, and normalization was done by GAPDH. Results are presented here as the representative of at least three independent experiments.

It was clear that miR-29b is a negative regulator of TRIM44 and CCNE1. The revelation that RV suppresses miR-29b directly pointed out to the involvement of its targets, i.e., TRIM44 and CCNE1 in RV pathogenesis. Time-kinetics of the protein expressions of TRIM44 and CCNE1 were analyzed (Figure 6A). With the progression of infection, as indicated by NSP1 protein levels, CCNE1 protein expression increased through 3, 6, and 9 hpi and was found to be maximum during 6–9 hpi with a subsequent decrease at 12 hpi. TRIM44 was also observed to be expressed progressively with RV infection, although maximally expressed at 12 hpi when prominent infection was observed. GAPDH was used as an internal control. The same samples were checked for the expression of the protein markers of the EMT pathway (Figure 6B). The loss of E-cadherin and the acquisition of N-cadherin expression in epithelial cells are indicative of activation of the EMT pathway (Mrozik et al., 2018; Loh et al., 2019). Time-kinetics of the EMT pathway protein markers reveal the loss of E-cadherin with the progression of infection and increased protein expression of N-cadherin compared to the Mock-infected Caco2 cells. Snail, a transcription factor, which triggers the EMT pathway was elevated in RV-infected cells as compared to mock-infected cells. Another transcription factor, Slug of the same family, showed a delayed elevation at 9 hpi. In the light of our results, we infer that TRIM44 and CCNE1 are significant in triggering the EMT pathway during RV infection, a phenomenon that may be required for cell-to-cell spread of RV to expand its infection within the host.

### miR-29b Overexpression Is Efficient in Diminishing Rotavirus Replication

**FIGURE 7:**
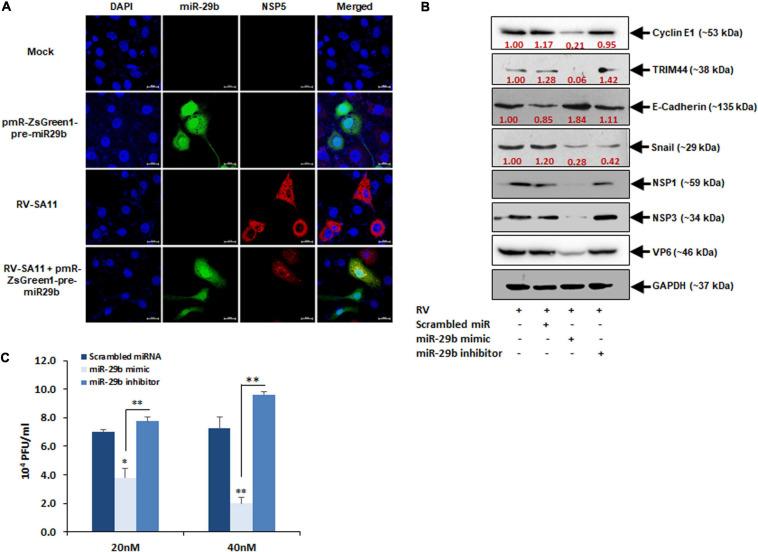
Overexpression of miR-29b inhibits RV replication. **(A)** MA104 cells were transfected with Pre-miR-29b construct cloned in pmR-ZsGreen1 mammalian expression vector followed by RV-SA11 infection. Cells were then fixed, permeabilized at 6 hpi, and stained with antibody against NSP5 to detect virus infection (red) and expression of miR-29b (green). Scale bars: 20 μm. **(B)** Caco2 cells were transfected with scrambled-miR, mimic miR-29b, or anti-miR-29b (40 nM each) for 48 h followed by infection with RV-SA11. The expressions of cyclin E1, TRIM44, E-Cadherin, and Snail were examined by immunoblot analysis at 6 hpi. NSP1, NSP3, and VP6 expressions were analyzed in the same set of samples to examine the level of virus infection. GAPDH was used as an internal control for obtaining the expression levels after normalization. These results are presented as the representative of at least three independent experiments. **(C)** Cells were transfected with scrambled-miR, mimic, or anti-miR-29b for 48 h followed by RV infection. Infectious virus particle was measured at 6 hpi by virion quantification assay. This result is presented as the mean and standard deviation from four experimental replicates. ^∗^*p* ≤ 0.05; ^∗∗^*p* ≤ 0.01.

As RV infection has been found to suppress miR-29b expression, it would be interesting to note the changes in RV replication should we overexpress miR-29b in epithelial cells. The pmR-ZsGreen1-pre-miR-29b construct was transfected into the MA104 cells and then infected with RV-SA11 for 6 h and prepared to be stained with an antibody against NSP5 for virus detection. A decrease in virus infection, indicated by anti-NSP5 (red) in the presence of miR-29b overexpression (green), was witnessed as compared to only RV-SA11 infected cells (Figure 7A). This was further clarified through the miR-29b mimic/inhibitor transfection assays; 40 nM of scrambled miR, miR-29b mimic, or miR-29b inhibitor-transfected Caco2 epithelial cells was RV-SA11 infected for 6 h and analyzed for the protein expressions of miR-29b targets and EMT pathway markers (Figure 7B). NSP1, NSP3, and VP6 protein expression levels symbolized RV infection. The protein expression levels of CCNE1 and TRIM44 were significantly decreased in miR-29b mimic-transfected, RV-infected cells compared to the only RV-infected cells. In compliance with these results, we found that the protein expression levels of E-cadherin had restored, whereas the expression of Snail had decreased in miR-29b mimic-transfected, RV-infected cells which specify that the EMT pathway was suppressed by the miR-29b mimic. However, these results were reversed in miR-29b inhibitor-transfected, RV-infected cells representing the similar effects of miR-29b inhibitor and RV infection on epithelial cells. Since the expression of the viral proteins NSP1, NSP3, and VP6 was decreased in miR-29b mimic-transfected cells, we could conclude that miR-29b is capable of suppressing RV replication and pathogenesis in epithelial cells. To confirm this, we also quantified the number of plaque-forming units (pfu) in the 20 nM miR-29b mimic-transfected, RV-infected cells, which was decreased by half as compared to the scrambled-miR transfected or miR-29b inhibitor-transfected, RV-infected cells (Figure 7C). The RV titer was further decreased with an increase in the miR-29b mimic to 40 nM. The suppression of RV replication in an miR-29b mimic dose-dependent manner was convincing enough to suggest that miR-29b is an efficient suppressor of RV infection.

## Discussion

The findings from our study could be summarized as follows: (1) miR-29b is transcriptionally regulated by RV through NSP1–p53 interaction, (2) miR-29b expression is crucial in the regulation of the EMT pathway through TRIM44 and CCNE1, a pathway required by RV to establish its pathogenesis in the host, and (3) miR-29b is capable of decreasing RV replication and infection in the host. As per our knowledge, this is the first report to highlight the direct role of miR-29b in reducing RV replication and RV infection spread through the EMT pathway.

Downregulation of miRNAs has been observed in many bacterial infectious diseases such as those caused by *Helicobacter pylori* (Xie et al., 2017), *Listeria monocytogenes* (Ma et al., 2011), *Salmonella* spp. (Schulte et al., 2011) parasitic, for example, those caused by *Leishmania* spp. (Ghosh et al., 2013; Geraci et al., 2015; Fernandes et al., 2019), as well as viral infectious diseases such as HCV (Mukherjee et al., 2014; Mukherjee et al., 2015; Li et al., 2017), hepatitis B virus (HBV) (Wang et al., 2012; Gao et al., 2015), human papillomavirus (HPV) (Shishodia et al., 2014), herpes simplex virus (HSV)-1 (Pan et al., 2019), transmissible gastroenteritis coronavirus (Ma et al., 2018), and SARS-human coronavirus (Leon-Icaza et al., 2019). miRNA downregulation has been noted in RV infection as well. Cellular miRNA, miR-525-3p has been known to be downregulated in RV infection, the mimic of which has anti-viral properties. This miRNA could decrease RV replication directly by targeting RV-NSP1 and also indirectly by facilitating the induction of the type I IFN pathway (Tian et al., 2017). In another study, RV-NSP5 was identified as a key modulator of miR-142-5p-mediated EMT in TGFβ-stimulated microsatellite stable colorectal cancer cells (Chanda et al., 2016). Earlier, our group also investigated that suppression of let-7g and upregulation of miR-99b to be crucial in the induction of autophagy that in turn facilitates RV infection, suggesting let-7g and anti-miR-99b as potential antirotaviral (Mukhopadhyay et al., 2019). Our findings of RV-induced miR-29b downregulation (Figure 1) are in compliance with another study where miR-29c downregulation is observed in RV-induced biliary atresia in mice (Wang et al., 2016). The maximum suppression of miR-29b at 9 hpi (Figures 1A,B) marks that induction of the EMT pathway is crucial during the early phases of infection when it is primal for the virus to infect as many cells as possible to take over the host.

Rotavirus produces six non-structural proteins after infection in its host. NSP1 is one of the six non-structural proteins (NSP1–NSP6) and is capable of suppressing the innate immune responses of the host to cause a proficient infection. Although it had been known that NSP1 is capable of inducing the degradation of IFN regulatory factor, or IRF3 and β-transducin repeat-containing protein or β-TrCP to modulate the innate immune mechanisms (Barro and Patton, 2007; Qin et al., 2011), its role in the degradation of p53 during RV infection was eventually known in the later years (Bhowmick et al., 2013). The participation of NSP1 was confirmed in our study by A5-16 strain (Figures 2A,B). A5-16 is not a replication-deficient mutant of A5-13, instead, the N-terminus of the NSP1 in the A5-16 has a 500-nucleotide deletion, succeeded by a non-sense codon that makes the A5-16 NSP1 non-functional (Taniguchi et al., 1996; Bagchi et al., 2010). The mutated region in the NSP1 of A5-16 contains the RING domain of NSP1, indicating the significance of the RING domain in NSP1-mediated miRNA suppression, though it was clear that the entire NSP1 protein is required to mediate this suppression (Figure 2C). The inhibitory effect displayed by the deletion mutants of miR-29b promoter in the presence of RV-NSP1 also indicated the transcriptional suppression of miR-29b during RV infection (Figure 3). p53 is a key activator of the apoptotic pathway and is targeted by viruses, such as HCV, HPV, adenovirus, etc., in order to modulate apoptosis and the cell cycle in the host (Querido et al., 2001; Lan et al., 2002; Bernard et al., 2011; Nailwal et al., 2015). Similarly, the NSP1 protein of RV has been reported to target the p53 to proteasomal degradation to block the apoptotic pathway during the initial stages of RV infection. This inhibition could be released during the late infection stage, resulting from the diminished interaction between p53 and NSP1. p53 acts as a transcription factor to bind to the regulatory elements of the stress-responsive genes and, hence, requires a DNA-binding domain to do so. The proteasomal degradation results in the destruction of the DNA-binding domain such that p53 is unable to bring about the transcription of the pro-apoptotic genes (Bhowmick et al., 2013). Our *in silico* studies led us to the possibility of three p53-binding sites to be present at the cis-regulatory elements in the miR-29b gene (Figure 4A). Owing to the ubiquitin ligase properties of NSP1, it is possible that NSP1 directly interacts with the DNA-binding region of p53 to induce a proteasome-dependent degradation (Bhowmick et al., 2013) to block the p53-mediated transcriptional regulation of miR-29b observed in our study (Figure 4). Also, MG132, an inhibitor of the ubiquitin-tagged protein degradation, could stabilize p53 and release the suppression on miR-29b (Figure 4F). Inability of the pCMV-Δcore/C-p53 construct to repress miR-29b substantiates that the interaction between NSP1 and p53 takes place at the core/DNA-binding domain of p53, which is not available for NSP1 to degrade in the pCMV-Δcore/C-p53 construct (Figure 4G). Consequently, with a halt in the p53-mediated transcription of miR-29b, the RV infection progresses in the host. Similar reports of disruption in the DNA-binding domain of p53 to augment viral infection in the host have been remarked in the case of Epstein–Barr virus (EBV) and high-risk HPV infections (Sato et al., 2009; Martinez-Zapien et al., 2016).

MicroRNA-29b directly regulates the gene transcription of CCNE1 and TRIM44 by targeting their 3′UTRs (Figure 5). Cyclins along with cyclin-dependent kinases (CDKs) are absolutely crucial for cell cycle regulation. For example, Cyclin E pairs up with CDK2 to initiate DNA entry into the S-phase and replication (Fang et al., 2016). Thus, dysregulation of CCNE ends up making a cell cancerous. CCNE1 is a target of the Wnt signaling (Lin et al., 2020). Wnt/β-catenin signaling pathway downregulates E-cadherin and upregulates transcription factors such as SNAI1 and SNAI2 to stimulate the EMT or the EMT pathway that allows an epithelial cell to transform into a mesenchymal cell (Kalluri and Neilson, 2003; Kalluri and Weinberg, 2009; O’Leary et al., 2018). TRIM44, a member of the TRIM protein family, is an efficient regulator of carcinogenesis and its overexpression is characteristic of facilitated cell proliferation and cancer metastasis (Kashimoto et al., 2012; Zhu et al., 2016; Yamada et al., 2017). The cell invasive and migratory properties of TRIM44 have been attributed to EMT in situations of hepatocellular carcinoma (Zhu et al., 2016) and human esophageal cancer (Xiong et al., 2018). These reports support our findings of CCNE1 and TRIM44 being involved in the regulation of the EMT pathway (Figure 6). CCNE1 expression is specific for the DNA replication phase of the cell cycle, and therefore, its expression is maintained only for a short interval, i.e., up to 9 h, whereas TRIM44 being involved in the cell invasion and migration stages begins to express in the later hours.

Snail and Slug, the transcriptional repressors of the adhesion molecules, are critically involved in the induction of the EMT pathway. Encoded by the SNAI1 and SNAI2 genes, respectively, Snail and Slug directly bind to the E2 box-type elements in the promoter region of the E-cadherins to suppress its transcription (Nieto, 2002). The repression of E-cadherin was observed 6 h of post-RV infection when the expression of Snail was heightened. However, the expression of Slug was less at this time point (Figure 6). Although both Snail and Slug act as repressors of E-cadherin thereby triggering the EMT pathway, they have been known to express reciprocally to maintain an optimum regulation of the involved signaling cascades to promote cell invasion (Ganesan et al., 2016; Nakamura et al., 2018). Also, the expressions of CCNE1 and TRIM44 were elevated (Figure 6). In this case, it could be interpreted that the expression of Slug is increased eventually after the expression of Snail has decreased to prolong the repression of E-cadherin and stabilize the expression of N-cadherin to maintain the EMT pathway as the RV-infection progresses with time. The downregulation of Slug can also be accredited to the phosphorylation by CCNE1-CDK2 that causes ubiquitination leading to proteasomal degradation of Slug (Wang et al., 2015). RV is capable of inducing G1/S transition in epithelial cells (Bhowmick et al., 2014). The Slug protein is strictly regulated temporally as the cell cycle progresses. It is increasingly accumulated at the G1 and late S phases and is maintained at a lower level during the G1/S transition and mitosis, hence the reciprocity in the expression levels of CCNE1 and Slug (Wang et al., 2015). As clarified by the decrease in the viral protein expression adjacent to the decrease in Snail, TRIM44, and CCNE1 (all activators of the EMT pathway) due to the introduction of miR-29b mimic (Figure 7), it is evident that the EMT pathway is important to the spread of the RV infection. Thus, its prolongation is an added advantage to the virus. miR-29b is capable of reducing virus proliferation in the RV-infected epithelial cells (Figure 7). It is clearly an inhibitor of the EMT pathway, which complies with another study, where overexpression of miR-29 could alleviate fibrosis by inhibiting the EMT pathway (Hand et al., 2012). Although it is new to destroying RV, its importance in decreasing migration of cancerous cells has been well known (Yan et al., 2015; Kwon et al., 2018).

**FIGURE 8:**
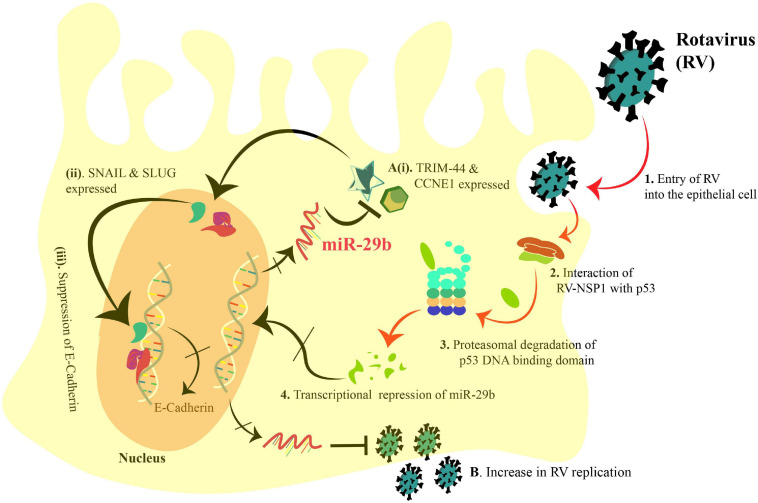
Schematic diagram representing the transcriptional suppression of miR-29b to induce the EMT markers and increase RV replication in an epithelial cell. 1. RV enters the epithelial cell, and 2. interacts with p53 via NSP1. 3. Proteasomal degradation of the p53 DNA-binding domain incapacitates p53 and leads to 4. transcriptional repression of miR-29b. **(A)(i)**. Thus, the miR-29b targets, TRIM44 and CCNE1, are expressed and **(ii)**. lead to the expression of Snail and Slug (EMT markers). **(iii)**. Snail and Slug lead to repression of E-Cadherin transcription leading to epithelial–mesenchymal transition. **(B)**. Also, the transcriptional repression of miR-29b facilitates the RV replication. Arrows denote upregulation, and blunt arrows denote inhibition. The crossed arrows indicate the suppression of the events. The black arrows indicate the findings of our study as mentioned in this manuscript.

In summary, we could identify the direct anti-viral role of miR-29b in decreasing the RV replication in the epithelial cells and while doing so, unraveling its significance in the EMT-mediated viral spread (Figure 8). Our study proposes miR-29b as a potent anti-viral against RV infection. It also opens new possibilities to consider miR-29b-targeted delivery to the gastro-intestinal epithelial cells to obstruct the viral dissemination.

## Data Availability Statement

The original contributions presented in the study are included in the article/Supplementary Material. Further inquiries can be directed to the corresponding author/s.

## Author Contributions

UM conducted the investigation and acquired the data. AB analyzed and interpreted the data and drafted the manuscript. MC-S revised the article critically for important intellectual content. AM conceptualized and designed the study, was in charge of the data curation, acquired funding, and supervised the study. All authors approved the final manuscript.

## Conflict of Interest

The authors declare that the research was conducted in the absence of any commercial or financial relationships that could be construed as a potential conflict of interest.
