# Supplementary material for: Rotavirus Induces Epithelial–Mesenchymal Transition Markers by Transcriptional Suppression of miRNA-29b
Source: Front Microbiol. 2021 Feb 18;12:631183. doi: 10.3389/fmicb.2021.631183 (PMC7930342; doi:10.3389/fmicb.2021.631183)
Supplement: Supplementary file 1 [file Table_1.DOCX]

| No | Oligo Name | 5'<-----Sequence----->3' |
| --- | --- | --- |
| 1 | **TRIM44-F_qSYBR** | **GAAGTTCCTCAGTCACCATCTG** |
| 2 | **TRIM44-R_qSYBR** | **TTCTATCTCCCTCTCCTGCTC** |
| 3 | **CCNE1-F_qSYBR** | **GATCCCCACACCTGACAAAG** |
| 4 | **CCNE1-R_qSYBR** | **CTCTATTTGCCCAGCTCAGTAC** |
| 5 | **RV-VP6-F_qSYBR** | **GCACAGCCATTCGAACATCATGC** |
| 6 | **RV-VP6-R_qSYBR** | **TGCATCGGCGAGTACAGACTC** |
| 7 | **RV-NSP4-F_qSYBR** | **GGATCCAGGAATGGCGTATTT** |
| 8 | **RV-NSP4-R_qSYBR** | **TTCTAGCTGGCGTCTCATTTC** |
| 9 | **GAPDH-F_qSYBR** | **AATCCCATCACCATCTTCCAG** |
| 10 | **GAPDH-R_qSYBR** | **AAATGAGCCCCAGCCTTC** |
| 11 | **3’UTR TRIM44wt_Mlu1-F:** | **ATATACGCGTCTAGGTTAGGATTGTCCT** |
| 12 | **3’UTR TRIM44mt_Mlu1-F:** | **TACGCGTCCTATCTCTGCAAAGACTGG** |
| 13 | **3’UTR TRIM44_Hind3-R:** | **TCTGAAGCTTAAATTTTTTCGAGAGTGGCC** |
| 14 | **3’UTR CCNE1wt_Mlu1-F:** | **AAGAACGCGTCTTCCACAACAGAAGTAT** |
| 15 | **3’UTR CCNE1mt_Mlu1-F:** | **CTACGCGTCCTAAGGGACTCCCACAACA** |
| 16 | **3’UTR CCNE1_Hind3-R:** | **GCACAAGCTTTGCCCGCAACCACCT** |
| 17 | **P0-29b-1500_Mlu1-F** | **ACTACGCGTCAGTTCTTAAATACAAGCC** |
| 18 | **PX-29b-936_Mlu1-F** | **ATTACGCGTCCTGGGTACATGAAGTATC** |
| 19 | **P1-29b-690_Mlu1-F** | **CCGGACGCGTCATTAGCATCTCATTAGT** |
| 20 | **P2-29b-423_Mlu1-F** | **CTCTGACGCGTCCTGCCTTTACCCAAG** |
| 21 | **P3-29b-300_Mlu1-F** | **ACTTACGCGTAAGGGAGTCCCA** |
| 22 | **P0-3-29b_Hind3-R** | **AGTGCAGCGAAGCTTGTCTCCCCCAA** |
| 23 | **pre-miR-29b_Xho1_F** | **CTCGAGTTCTCACAGCCATCCTGCTGTTGC** |
| 24 | **pre-miR-29b_Kpn1_R** | **GGTACCTCACAGCTAGGTTTCAACTTTTCC** |

**Rotavirus Induces Epithelial-Mesenchymal Transition Markers by Transcriptional Suppression of miRNA-29b**

**Supplementary data 1: Sequences of different primers used in this study.**


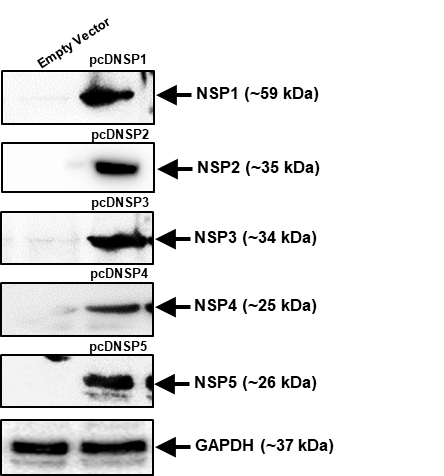


**Supplementary data 2: MA104 cells were transfected individually with the nonstructural proteins of RV NSP1-5 cloned in pcDNA6B. The transfection efficiencies were analyzed by immunoblot analysis at 48 hours post-transfection.**

**
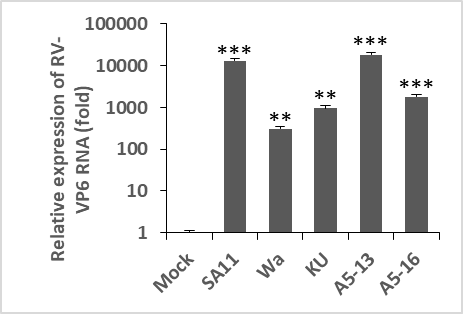
**

**Supplementary data 3: Caco2 cells were infected with different RV strains SA11 or Wa or KU or A5-13 or A5-16 or kept mock infected. RV-VP6 RNA level was measured and plotted as relative RNA level in comparison to mock-infected cells after normalization to the expression of GAPDH.**


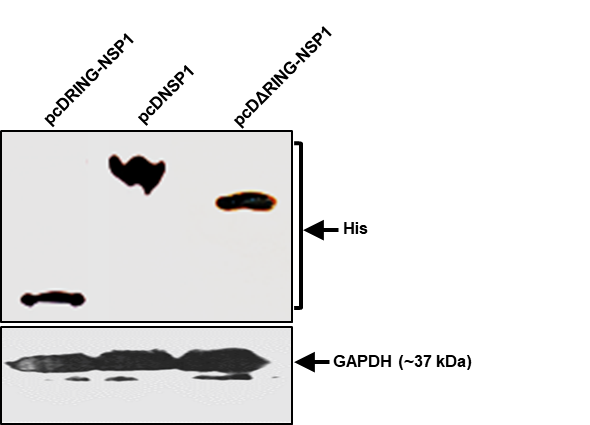


**Supplementary data 4: MA104 cells were transfected with the plasmid DNA encoding full length RV-NSP1 protein and its truncated mutants, pcDRING-NSP1 and pcDΔRING-NSP1, for 48 h, followed by immunoblotting with anti-His and anti-GAPDH antibody.**


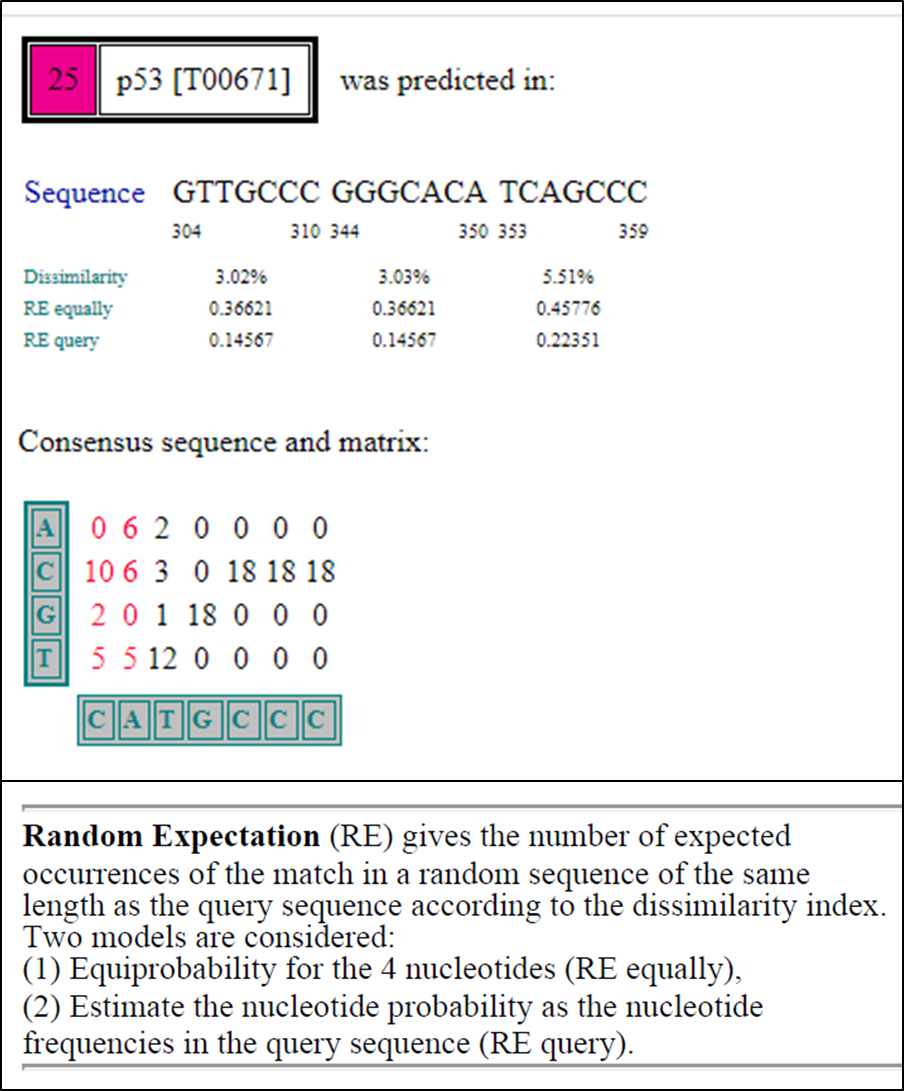


**Supplementary data 5: Screenshot image of predicted p53 binding sites/sequences between -423 to -300 upstream of pri-miR-29b transcription initiation site. [PROMO 3.0: Study of transcription factor binding sites in DNA sequences]**
